# Availability of tuberculosis infection control plans at rural hospitals of Vhembe district, Limpopo Province of South Africa

**DOI:** 10.4102/phcfm.v5i1.480

**Published:** 2013-06-26

**Authors:** Takalani G. Tshitangano, Sonto M. Maputle, Lizzy M. Netshikweta

**Affiliations:** 1 Department of Public Health, University of Venda, South Africa; 2 Department of Advanced Nursing Science, University of Venda, South Africa

## Abstract

**Background:**

In Limpopo province the rate of new tuberculosis (TB) cases increase daily. The Infection Control (IC) plan is one of the essential actions for TB IC. This study aimed to establish the availability of these plans at health care facilities.

**Objectives:**

The objectives were to explore and describe the awareness and knowledge of health care workers (HCWs) of the availability and content of TB IC plan; and to identity the role of infection control committees from the perspective of HCWs.

**Method:**

A qualitative approach using a cross-sectional descriptive design was adopted. The target population was all HCWs from the seven hospitals of Vhembe district. A purposive sampling approach was used to select 57 participants. The approval to conduct this study was obtained from the relevant authorities and participants. Data was collected through seven focus group discussions comprising five to 10 members. An unstructured discussion guide was used to collect data, and an open-coding method was used to analyse the data. Lincoln and Guba's criteria ensured trustworthiness of the study findings.

**Results:**

Findings revealed that HCWs were not aware of the availability and the information contained in the TB IC plans. No person was designated as TB IC officer at hospital level. There was lack of a TB IC Committee and teams as well as ineffective utilisation of those that did exist.

**Conclusions:**

It was concluded that if the TB IC plans are not available at health care facilities, then the TB IC practices implemented by HCWs vary, resulting in TB nosocomial infection transmission. It was recommended that the World Health Organisation's TB IC plans be adopted and implemented in Vhembe district.

## Abstrait

**La disponibilité de plans de contrôle de l'infection par la tuberculose dans des hôpitaux du district de Vhembe, dans la province sud-africaine du Limpopo**

**Contexte:**

Dans la province du Limpopo, le taux de nouveaux cas de tuberculose augmente quotidiennement. Les plans de contrôle de l'infection (CI) sont l'une des principales actions associées au contrôle de l'infection par la tuberculose. L'objectif de cette étude est de déterminer si ces plans sont disponibles dans les structures de soins.

**Objectifs:**

Cette étude avait pour objectif d’étudier et de décrire la sensibilisation des personnels soignants à la disponibilité et au contenu des plans de contrôle de l'infection par la tuberculose, ainsi que la compréhension de ces plans par ces derniers. L'objectif était également d'identifier le rôle des comités de contrôle de l'infection du point de vue des personnels de santé.

**Méthode:**

Une approche qualitative, descriptive et transversale a été adoptée. La population ciblée se composait de l'ensemble du personnel soignant des sept hôpitaux du district de Vhembe. Une approche par échantillonnage dirigé a été utilisée afin de sélectionner 57 participants. Les autorités pertinentes et les participants ont donné leur consentement à la réalisation de cette étude. Des données ont été recueillies au moyen de sept discussions de groupe, ces groupes se composant de cinq à 10 membres. Un guide de discussion non structurée a été utilisé afin de recueillir des données, et une méthode de codage ouvert a été utilisée afin d'analyser les données. Les critères de Lincoln et Guba ont permis de garantir la fiabilité des conclusions de l’étude.

**Résultats:**

Les conclusions ont révélé que les personnels de santé n’étaient pas informés de la disponibilité des plans de contrôle de l'infection par la tuberculose et des informations qui y figuraient. Personne n’était désigné responsable du contrôle de l'infection par la tuberculose au niveau de l'hôpital. Les comités et équipes de contrôle de l'infection par la tuberculose faisaient souvent défaut, et lorsqu'ils existaient, n’étaient pas utilisés de manière efficace.

**Conclusions:**

L’étude a conclu que lorsque les plans de contrôle de l'infection par la tuberculose faisaient défaut dans les centres de soins, les pratiques de contrôle de l'infection par la tuberculose mises en œuvre par les personnels de santé variaient, résultant sur une transmission nosocomiale de la tuberculose. L’étude a recommandé que les plans de contrôle de l'infection par la tuberculose de l'Organisation mondiale la santé soient adoptés et mis en œuvre dans le district de Vhembe.

## Introduction

In 2005, tuberculosis (TB) was declared an emergency by all Health Ministers from the African region who were gathered in Maputo attending the World Health Organization Regional Office for Africa (WHO/Afro) committee meeting. Every member state, including South Africa, was then asked to develop and implement a TB plan that would help to improve TB detection and the treatment success rate and reduce TB patients’ defaulter rates.^1^

Since time immemorial, TB has affected many lives and has resulted in the death of millions. In 2011, 8.7 million new cases of TB were reported. Of these, about 1.4 million people succumbed to the disease and died.^2^ It was discovered then by the World Health Organization (WHO) that tuberculosis is one of the top killers of women.^2^ Nationally, the average death rate in 2010 was at 6.5%. In Limpopo Province, the death rate was higher than the national average and was at 8.5% in 2010.^3^ Limpopo province was the second highest province, with a death rate of 8.5%, as discussed at the third South African TB conference in 2012.^4^

One of the goals of the National Strategic Plan of South Africa (NSPSA) for 2012–2016, the Millennium Development Goal (MDG) number 6 and the Stop TB partnership targets of WHO, is to reduce by 500 the number of new TB infections as well as deaths due to TB by 2015.^2^ The reduction of new infections and deaths due to TB can only be possible if facilities could avail themselves of and implement the proposed TB Infection Control (TB IC) plans.^4^

The Indian Ministry of Health and Family welfare^5^ states that TB IC plans serve to establish visible commitment of the facility to prevent infection transmission. The TB IC plans articulate clear policies and procedures to ensure proper implementation and make staff roles and responsibilities clear.^6^ According to the WHO^6^, the development of an Infection Control (IC) plan is one of the essential actions for effective TB infection control.

The WHO^7^ believes that TB IC plans set aims and goals, compliance requirements, roles, responsibilities, policies, and procedures to administer and manage a TB infection control programme. According to the WHO,^7^ the TB IC plan identifies TB transmission risk settings in a facility and makes TB information available to health care workers (HCWs) and patients. The TB IC plan prescribes the measures to be implemented in a given facility to control TB infection. The WHO^8^ also emphasises that TB IC plans sets out standards for early recognition, adoption and initiation of airborne precautions to be implemented in either suspected or confirmed TB patients. According to the WHO,^9, 10^ the TB IC plan formalises the establishment of TB IC committees as well as the appointment of one individual per facility who is assigned the duty and authority to ensure that the prescribed infection control measures are adhered to.

According to the WHO,^6, 11^ every health care facility which renders TB services to suspected or confirmed TB patients should develop and implement a TB IC plan. The plan outlines standards for early recognition, adoption and initiation of airborne precautions implemented in suspected or confirmed TB cases.^12, 13^

### Problem statement

Despite the wide coverage and adoption of the Directly Observed Treatment Short course (DOTS), which has saved millions from the scourge of TB worldwide, the TB case load in the Limpopo province has been increasing from 11 897 in 2005 to 22 158 in 2011.^14^ The number of TB cases in Vhembe district for the year 2009 was 2194. Of these, 750 cases were women of child-bearing age (aged 15–44 years). About 20 were children from 0–4 years of age.^15^ Pakenham-Walsh and Bukachi^16^ associate the lack of TB IC plans with lack of access to the basic, practical information and direction needed to ensure safe and effective TB care_._ There is currently no information that describes the availability of TB IC plans in South Africa as a whole, and Limpopo province and Vhembe district, more specifically for this study. It is not clear whether such plans are available or not in rural hospitals. This study, therefore, was conducted in order to investigate the availability of TB IC plans at the rural hospital of Vhembe district in order to;

- explore and describe the awareness of health care providers regarding the availability of TB IC in the hospital
- assess the knowledge of health care providers on the content of the TB IC plan
- identify the role of infection control committees from the perspective of health care providers.

The results will serve as baseline information upon which the Department of Health (Limpopo province and Vhembe district) will plan and conduct workshops in order to empower TB managers and TB teams with information regarding how to develop TB IC plans and determine their contents.

## Ethical considerations

The Limpopo Provincial Department of Health approved the study, and ethical clearance was issued by the University of Venda, Research Ethics Committee. Permission to access the seven hospitals was obtained from each hospital manager. The participants signed informed consent, and anonymity and confidentiality were observed throughout the study.

## Research method and design

### Study approach and design

This study adopted a qualitative approach using a cross-sectional descriptive design.^17, 18^ A case study method of inquiry was used.^17^

### The study setting

The study was conducted in the district of Vhembe, in the Limpopo province of South Africa. Vhembe district is situated in the far-northern side of South Africa, bordered by Zimbabwe and Mozambique, and consists of four subdistricts, namely, Thulamela, Makhado, Mutale and Musina. This district has seven hospitals, 112 clinics and eight health centres. The study was conducted at the seven hospitals of Vhembe district.

### The target population

The target population of this study was all types of HCWs in the participating hospitals. The study hospitals differed in size in terms of number of beds and the number of wards. Some hospitals had only three wards, whereas others had a maximum of nine. The researchers were satisfied to welcome one representative from each of the wards per study hospital.

As a result, 57 HCWs participated in this study, as described in Table 1.

**TABLE 1:** Breakdown categories of participants.

Category and unit	Number
Deputy manager	1
Laboratory staff	7
Surgical ward nurses	4
Antiretroviral therapy clinic nurses	2
TB focal point personnel	2
Paediatric ward nurses	7
Outpatient department/Casualty nurses	7
X-ray unit staff	7
TB ward nurses	5
Medical ward nurses	7
Infection control nurses	3
Occupational Health and Safety nurse	1
Pharmacy staff members	2
Subacute ward nurse	1
Maternity ward nurse	1
Psychiatric ward nurse	1

**Total**	**57**

*Source*: Data collected during study

### Data collection methods

Data for this study was collected through focus group discussions (FGDs), with HCWs serving as focus group members. There was only one focus group per study hospital, making seven focus groups in seven hospitals, each comprising five to 12 participants, depending on the size of the hospital in terms of the number of wards.

### Data collection tool

One unstructured question, namely, ‘Could you please describe what is contained in the TB IC plan implemented in this hospital?’ was used to collect data from the participants. This question was followed by probing questions that needed clarity regarding the availability of the TB IC plan, IC officer and IC Committee or team, as well as what the TB IC plan of the hospital covered. Each focus group session lasted between one hour thirty minutes and two hours to enable the researcher to obtain the necessary information until theoretical saturation of each category was reached.^18^

### Data collection process

The researcher set the date and time for the FGDs and communicated these to the hospital managers, requesting help regarding the arrangement of the venue and delegation of representatives. On the set date and time, the researcher found the participants assembled in venues arranged by the managers. The participants comprised representatives from each unit of the hospital. The researcher facilitated FGDs of between five to 12 participants. Permission to use the voice recorder to capture the information accurately was negotiated with the participants. Only one FGD session per participating hospital was facilitated.

### Data analysis

Analysis of the data was guided by Tesch's eight–step open-coding method as discussed in Cresswell,^19^ where the researcher read through all of the field notes (from the FGDs) and interpreted them carefully in order to get a sense of the entire notes. The interpretations were written as themes and similar themes were clustered together and arranged finally into one major theme and four subthemes. The tape-recorded information served as a back-up.

## Trustworthiness

Trustworthiness was ensured through the use of Lincoln and Guba's criteria^20, 21^ as outlined in Creswell,^19^ namely, credibility, transferability, confirmability and dependability. Credibility was ensured by prolonged engagement of the participants in FGDs lasting from one-and-a-half to two hours. Triangulation of data capturing was performed through the use of the voice recorder during FGDs and the writing of field notes. Referential adequacy using a voice recorder to capture the interviews provided a suitable authentic record.^22, 23^ Tape recordings as well as field notes taken during FGDs increased the confirmability of the research. Transferability was ensured by a complete description of research method and interpretation of the research findings in the study report.

## Results

In the field notes, the seven hospitals were given codes in the form of letters of alphabet (A–G) to ensure their privacy and anonymity. The main theme and four sub-themes that emerged during data analysis are presented in Table 2.

**TABLE 2:** Theme and subthemes.

Theme	Subthemes
Availability of TB IC plans at seven rural hospitals of Vhembe district.	HCWs were not awareness of the availability of TB IC plans in their hospitals There was knowledge deficit regarding the content of TB IC plans No person was designated as TB IC officer in hospitals There was lack of TB IC committee and teams as well as ineffective utilisation thereof

*Source*: Data collected during study

### Infection control plans at rural hospitals

The tuberculosis IC plans establish goals, compliance requirements, roles, responsibilities, policies, and procedures to administer and manage a TB IC programme.^24, 25^ The TB IC plan further identifies high risk areas for TB transmission in a facility and provides TB information to HCWs and patients. TB IC policies, procedures and plans are measures to be implemented in a given facility to control TB infection. According to the WHO,^26^ one individual in a facility should be assigned the responsibility and accorded the authority to monitor the implementation of the TB IC plan. In this study, empirical data collected through FGDs revealed the subthemes as displayed in Table 2. Each subtheme is discussed individually below.

#### No awareness of infection control plans

The participants indicated that clients who present with TB symptoms are managed according to different protocols that are available in each of the health care facilities, and said that they were not aware of the WHO TB IC plans. They indicated that they were not aware that a TB IC plan existed at their hospitals, and the next paragraphs show what some of the participants said during the FGDs.

The TB ward nurse from hospital A said:

‘I am not aware of the TB IC plan of this hospital. I don't know the contents either’. (P1, Male, 48)

The OPD/casualty nurse from hospital G concurred, stating that:

‘I am not aware of any TB infection control plan of this hospital… what I know are these posters that are hanged on the walls’. (P2, Female, 33)

To further confirm the non-awareness of the availability of a TB IC plan, the laboratory technician from hospital A said:

‘I have never seen any TB IC plan in this hospital’. (P3, Male, 37)

A nurse working in the Isolation ward at hospital F said:

‘I am not aware of any TB plan … but the universal standard precautions are the only control measures I know of’. (P4, Female, 52)

The nurse working in the Pediatric ward at hospital B said:

‘I am not aware of TB IC plan of this hospital’. (P5, Female, 46)

The Casualty nurse from hospital E said:

‘I am not sure if the hospital does have an infection control plan’. (P6, Female, 43)

The OPD/casualty nurse from hospital C concluded by saying:

‘… the hospital does not have an infection control plan’. (P7, Female, 46)

#### Little or no knowledge regarding content

Although the participants were providing preventative and curative care to TB clients, when asked about the TB IC plans, variable and inaccurate information was obtained. Some of the focus group members misquoted dates of policies and the content was not known. The following paragraphs cover some of what was said by the participants during FGDs.

The nurse who was working in OPD at hospital F said:

‘I don't know the TB IC plan of the hospital. We manage TB patients using the policy for general infection control of the National Department of Health (2005) [*Date misquoted*], which I think is still a draft’. (P8, Female, 43)

The nurse who was working in the TB ward at hospital C said:

‘HCWs in the TB ward are guided by the National TB control programme (NTCP, 1996) [*date misquoted*]. Yaaa… we have policies that we use to manage, treat and monitor TB patients.’ (P9, Male, 47)

The most disturbing was when the TB focal point nurse from hospital C said:

‘I only know the National infection control policy (NICP, 2005), which is still a draft [*date misquoted*]. The content is like general infection control in the hospital setting’. (P10, Female, 46)

#### No infection control officer

The majority of the hospitals in Vhembe district did not have a person designated as a TB IC officer. Other participants indicated that quality assurance nurses and occupational health and safety nurses were delegated the roles of TB IC officers. The following statements from the participants have been cited to support the findings.

A radiographer from hospital G said:

‘The quality assurance nurse in this hospital is assigned the responsibility of managing infection control, including TB IC in the hospital’. (P11, Male, 38)

In contrast, the laboratory technician from said the same hospital said:

‘There is no dedicated infection control nurse in this hospital’. (P12, Male, 53)

Same sentiments were shared by a nurse who was working in the surgical ward from hospital B when she said:

‘This hospital does not have an infection control nurse. The Occupational Health and Safety nurse is responsible for infection control in the hospital’. (P13, Female, 41)

The situation was the same in hospital C, as stated by a nurse who works in the female medical ward:

‘The Occupational Health and Safety nurse has been assigned the responsibility of managing infection control in the hospital’. (P14, Female, 53)

#### Ineffective utilisation of available structures

Of the seven participating hospitals, only four indicated that their hospitals have infection control committees. However, functions of these committees were different amongst the hospitals. It was further discovered during the FGDs that the available committees were not utilised effectively. The general perception was that the committee would only focus on ordering protective clothing. Furthermore, it was determined that the terms ‘team’ and ‘committee’ were used interchangeably to mean a structure responsible for infection control in the hospital. The quotations in the following paragraphs are presented in support of the lack of and/or ineffective utilisation of TB IC committees (‘team’ and ‘committee’ used interchangeably).

The nurse who was working at psychiatric ward at hospital B said:

‘No, this hospital does not have an infection control committee’. (P15, Female, 48)

In contrast, the nurse who was working in the male medical ward in hospital C said:

‘What I know is that the hospital does have an infection control committee, which is not functional, because I don't know what their role is’. (P16, Male, 53)

The Deputy Manager from hospital F was also of the opinion that the committee is available but not utilised effectively, saying:

‘There is an infection control committee in this hospital, which is responsible for ordering and distributing protective clothing such as masks, gowns, gloves, goggles, boots and plastic aprons’. (P17, Female, 16)

The nurse working at the ARV clinic at hospital F said:

‘The infection control team is responsible for ordering and distributing protective clothing such as masks, gowns, gloves, goggles, boots and plastic aprons’. (P18, Female, 38)

## Discussion

The findings of this study indicated that not one of the hospitals in Vhembe district had a TB IC plan. The findings are supported by Sissolack, Marais and Mehtar^27^ in a study that was conducted to investigate factors influencing TB prevention and control practices from the perspective of nurses at Tygerberg Hospital in Cape Town, South Africa, which identified lack of a TB IC plan as being one of the factors associated with the risk of TB transmission in health care facilities. A similar study conducted by Luhalima, Netshandama and Davhana-Maselesele,^28^ to evaluate the implementation of tuberculosis policies at a regional hospital in the Limpopo province, revealed that a TB IC plan was not available in the wards and participants expressed lack of knowledge of such a plan.

Furthermore, the findings of this study concur with Pakenham-Walsh and Bukachi,^28^ who discovered that members of the health care team in developing countries continue to be deprived of basic, practical information, which would enable them to render safe and effective TB care. Pakenham-Walsh and Bukachi^16^ argue that gross deficiency of basic information on how to manage common diseases is often associated with substandard, often dangerous, health care practices and mismanagement of a lot of diseases. The analogy example to this is documented in Oluwole,^29^ where lack of information was found to be a barrier to breast self-examination in a study conducted to determine the level of awareness, knowledge and practices of breast self–examination in Nigeria. Similarly, another study cited in Oluwole^29^ stated that the prevention of suicide has not been adequately addressed worldwide due to a basic lack of information about suicide as a major problem. The WHO^9^ emphasises that lack of TB IC plans could lead to TB infection control through trial and error methods resulting from different perceptions to various practices of TB IC.

The WHO^26^ believes that a TB IC plan directs the path and practices of members of the health team in a health care setting, when dealing with suspected cases of TB. Thus, the effectiveness of TB IC measures is directed by a clear TB IC plan. Simple TB control measures can reduce nosocomial TB transmission amongst patients significantly when such measures are directed by a clear TB IC plan.^30, 31, 32^

## Practical implications

In order to ensure effective TB IC, every health care facility must develop and implement a TB IC plan. The TB IC plan should direct the path and practices of members of the health team in a health care setting, when dealing with suspected cases of TB. The TB IC committee should oversee TB infection control issues in a hospital setting, and the TB IC officer should monitor the implementation of the TB IC plan in this setting. When the TB IC plan is implemented effectively, contribution to the achievement of MDG Goal number 6 will be realised.

## Limitations

The study was only conducted at hospitals in Vhembe district, and the findings are therefore not transferable to all hospitals in the entire Limpopo province. However, these findings can serve as a basis upon which a decision can be made with regard to conducting a similar study on a wider scope such as the entire Limpopo province or South Africa as a whole.

## Conclusions

It was concluded that if the TB Infection Control plans are not available at the hospitals in Vhembe district, TB IC practices implemented by HCWs will vary greatly. As a result, TB infection transmission will not be reduced, which could theoretically be extrapolated as leading to the failure of the country as a whole with regard to the control of TB infection.

## Recommendations

The following recommendations were meant to address each of the study findings:

- The Department of Health, Vhembe district should, in collaboration with the University of Venda, organise a seminar or workshops where TB managers and their teams would be empowered on how to develop TB IC plans and their contents. Thereafter, each health care facility would be obliged to develop its own TB IC plan.
- Each hospital should then organise a follow-up workshop targeting all HCWs responsible for TB management in the hospital where the contents of the TB IC plans will be presented.
- Each hospital should, in response to the stipulations of the plan, appoint a TB IC committee as well as one or more TB IC officers.
- The TB IC officer should be assigned the responsibility of ensuring that the developed TB IC plan is implemented correctly.

The possible future direction of the study

A follow-up study should be conducted to assess the impact of developing and implementing TB IC plans on TB infection transmission at hospitals of Vhembe district.
